# Larva Migration and Eosinophilia in Mice Experimentally Infected With *Gnathostoma spinigerum*

**Published:** 2012

**Authors:** W. Saksirisampant, N. Choomchuay, K. Kraivichian, B. Wongsatayanon Thanomsub

**Affiliations:** 1 Dept. of Microbiology, Faculty of Medicine, Srinakharinwirot University, Bangkok, Thailand; 2 Dept. of Parasitology, Faculty of Medicine, Chulalongkorn University, Bangkok, Thailand; 3 Dept. of Pathology, Faculty of Medicine, Srinakharinwirot University, Bangkok, Thailand

**Keywords:** *Gnathostoma spinigerum*, Larvae, Worm, Larva migration, Eosinophil

## Abstract

**Background:**

*Gnathostoma spinigerum* causes larva migran in human which is endemic in Southeast Asia. Information regarding larva migration is limited. In this study, we investigated the parasite migration by recovery of worms from the whole body of mouse after oral infection with advanced third stage larvae (AL3). The percentage of blood eosinophils was examined in parallel.

**Methods:**

Mice were orally infected with AL3 and histological study of organs was investigated in order to study the migration of AL3, along with blood eosinophilia.

**Results:**

At 1 hr post infection (PI), the larvae remained in the stomach, thereafter at 3, 5, 7, 10 and 24 hr PI; they were recovered from various organs including liver, mesentery, esophagus, diaphragm, lung, heart and dorsal fat. At day 15 PI, they were mostly found in muscles (76.47%). The average worm recovery (5 months) was 78.03%. The worms were found in the liver at every time point. Larva encystment was detected. There was a significant difference in blood eosinophils between the 8 larvae- (average 9.33% + 6.25%) and the 15 larvae-infected groups (average 22.66% + 11.03%). Surprisingly, the blood eosinophils (average 19.00% + 2.92%) were not higher in the higher infective dose- group (25 larvae).

**Conclusion:**

Liver was involved by *G. spinigerum* throughout the study. We detected larva encystment which had never been reported in human gnathostomiasis. The highest percentage of eosinophil occurred during the invasive stage.

## Introduction

Gnathostomiasis is endemic in many countries of Southeast Asia. It has been also reported in parts of Mexico and certain countries in South America (1, 2). Seven species of *Gnathostoma* have been reported in human infection (2, 3). Globally, most common is *G. spinigerum* which is found in Thailand (2, 4). The worm is actually a nematode of animals (dog, cat, tiger, lion, leopard, boar, weasel, ferret, mink etc.) and humans are an accidental host (1, 4). Human infection is now also considered an emerging disease in Europe and America (3–6). Infection is caused by consumption of insufficiently cooked or raw fresh water fish containing advanced third stage larvae (AL3) (1, 7). Patients with this infection usually visit doctors with subcutaneous larva migrans, i.e. migratory swelling with pain and pruritus (7, 8). In addition, visceral larva can result in ocular involvement, cough, hematuria and others symptoms (9–13). The disease could also cause near fatal eosinophilic meningitis and/or meningoencephalitis (6, 14, 15). A heavy infiltration of eosinophils, fibroblasts and histiocytes, and mild to moderate edema of involved visceral organs has been observed in humans (16). The definite diagnosis of infection is based on worm recovery from a skin lesion or biopsy specimen. A presumptive diagnosis is based on history of raw and/or undercooked fresh water fish consumption, blood eosinophilia and a positive serological test. Serological tests can not differential between present and past infection (17–19). Blood eosinophilia is an important hallmark which can be an early indicator for the diagnosis and evaluation of the effectiveness of treatment (8, 20). Information regarding host-parasite interaction in terms of pathogenesis, immune response is still limited. Rodents have been used as animal models for studying the effectiveness of chemotherapy of this species and those of *G. hispidum* and *G*. *nipponicum* infections (21–26). Rodents are accidental hosts similar to humans (1).

We used the mouse model to study parasite migration by recovery of worms from the whole body after oral infection with AL3. The percentage of blood eosinophils was examined in parallel.

## Materials and Methods

### Experimental infection of mice with G. spinigerum

Advanced third stage larvae of *G. spinigerum* (AL3) were obtained from the liver of naturally infected eels (*Monopterus alba*) by digestion with artificial pepsin as previously described (27). We used adult male Swiss albino mice (8 weeks old, weighing approximately 34 g). They were fed with 8, 15 or 25 AL3 by gastric intubation. They were reared under a hygienic environment, in polycarbonate shoe-boxes in the animal house and fed ad libitum with food pellet and de-chlorinated tap water. Food was with-held for 3 hr prior to infection.

### Worm recovery, histology and blood eosinophil counts

Worms were recovered from thirty-three mice individually infected with 8 viable larvae each. At different time intervals they were sacrificed in order to study migration. The organs and muscles were separately removed, sliced and pressed between two thick glass plates, then examined under a stereo microscope. Tissues were fixed in formaldehyde and sent to pathology for processing, sectioning and staining with hematoxylin and eosin. Blood eosinophil counts were examined in three groups of mice infected with 8, 15 and 25 larvae as the percentage of total white blood cells. Blood smears were taken from the ophthalmic vein by using a microhematocrit tube and stained with Field's stain solution (28). They were also checked prior to the infection at Day 0. This study was approved by the Ethics committee dealing with animals of the Faculty of Medicine, Chulalongkorn University, Bangkok Thailand.

### Statistical analysis

Statistical significance was determined by Student- *t* test. A value of *P*<0.05 was considered to be significant different.

## Results

### Larva migration

The group of mice infected with 8 larvae each was used to study migration. All larvae were still found in the stomach at 1 hr post infection (PI). At 3 hr PI, the worms began migrating to liver (6.67%) and mesenteries (13.33%) but most were still in the stomach (80.00%). Thereafter, they moved to various organs including esophagus, liver, lung, heart and diaphragm (Table 1).

**Table 1:** Mean percentage of larvae recovery in various locations at different time intervals after infection with 8 larvae

Time Post infection	No. Mice studied	Total worm Recovered	No. worm recovered (%) and Location											
	
			St	Eos	Li	Mes	Lu	He	Di	Dfat	Mus	Uro	Sub	Br
1 hr	2	16 (100)	16 (100)	-	-	-	-	-	-	-	-	-	-	-
3 hr	2	15(93.75)	12 (80.00)	-	1 (6.67)	2 (13.33)	-	-	-	-	-	-	-	-
5 hr	2	11 (68.75)	1 (9.09)	2 (18.18)	3 (27.27)	-	2 (18.18)	1 (9.09)	2 (18.18)	-	-	-	-	-
7 hr	1	7 (87.50)	4 (57.14)	-	-	1 (14.28)	2 (28.57)	-	-	-	-	-	-	-
10 hr	1	7 (87.50)	6 (85.71)	-	1 (14.29)	-		-	-	-	-	-	-	-
24 hr	2	10 (62.50)	4 (40.00)	1 (10.00)	3 (30.00)	-	1 (10.00)	-	-	1 (10.00)	-	-	-	-
3 d	2	9 (56.25)	-	-	5 (55.56)	-	-	-	-	3 (33.33)	1 (11.11)	-	-	-
5 d	2	10 (62.50)	-	-	7(70.00)	-	-		1(10.00)		2(20.00)	-	-	-
7 d	3	11(45.83)	-	-	7(63.64)	-	-	-	-		4(36.36)	-	-	-
15 d	3	17(70.83)	-	-	1(5.88)	-	-	-	-	1(5.88)	13(76.47)	1(5.88)	1(5.88)	
1 Mo	4	26(81.25)	-	-	4(15.39)	-	-	-	-	-	21(80.76)			1(3.85)
3 Mo														
	5	37(92.50)	-	-	3(8.11)	-	-	-	-	-	31(83.78)		1(2.70)	2(5.40)
5 Mo														
	4	30(93.75)	-	-	1(3.33)	-	-	-	-	-	26(86.67)		1(3.33)	2(6.67)
Total	33	206(78.03)	-	-	-	-	-	-	-	-	-	-	-	-

Abbreviations: St; Stomach, Eo; Esophagus, Li; Liver, Mes; Mesentery, Lu; Lung, He; Heart, Di; Diaphragm, Dfat; Dorsal fat, Mus; Muscle, Uro; Urogenital organ, Sub; Subcutaneous tissue, Br; Brain

**Fig. 1:**
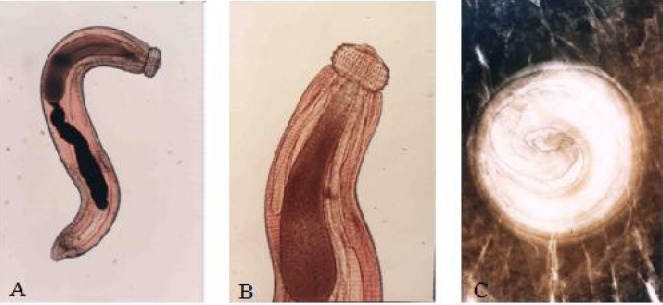
Image of *Gnathostoma spinigerum* advanced third stage larva (AL3) recovered A) Whole larva B) Close up of the anterior part/head bulb C) Encysted larva of *Gnathostoma spinigerum* recovered during 1 month PI from the invaded organs

During 3 hr and 24 hr PI, a few worms were still in the stomach. Then almost all migrated to other organs. At Day 5 PI, 70.00% of the larvae were still observed in the liver and first recovered in diaphragm (10.00%) and in the abdominal (20.00%). Noteworthy, larvae started to appear in the liver at 3 hr PI and could be observed at every time point after infection throughout the experiment. At Day 15 PI, the worms invaded subcutaneous tissue (5.88%). Of all the 33 infected mice, only 3 animals demonstrated subcutaneous involvement. At 1, 3 and 5 months PI, the larva could be found in the brain. However, no abnormality or death was observed prior to sacrifice. Morphology of migrated worms indicated that they were third stage larva with four rows of hooklets on the head bulb (Figs. 1A, 1B) (4).

**Fig. 2:**
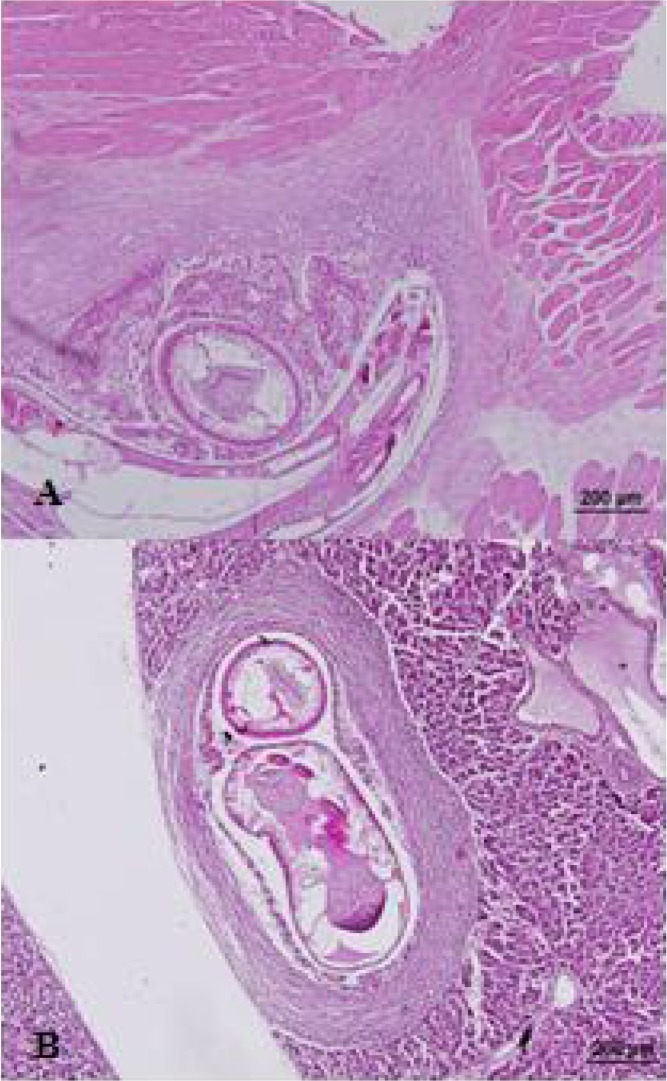
Histological sections of encysted *Gnathostoma spinigerum* larva in organs: A) Diaphragm at Day 20 PI, showing inflammatory cells infiltration B) Liver at 5 months PI, showing dense fibrous tissue surrounding the larva

The inflammatory cell infiltration around larva invading organs was first observed in Day 11 PI (Data not shown). The gross appearance of larvae encystments with one larva coiled inside one cyst was observed during Day 20 and one month PI as shown in Fig. 1C. Histological sections thereafter showed that the larvae were surrounded by thick fibrous tissue and chronic inflammatory cells, predominantly small lymphocytes seen in various organs for example, diaphragm, liver (Figs. 2A, 2B) and others (data not shown).

### Blood eosinophilia

**Fig. 3:**
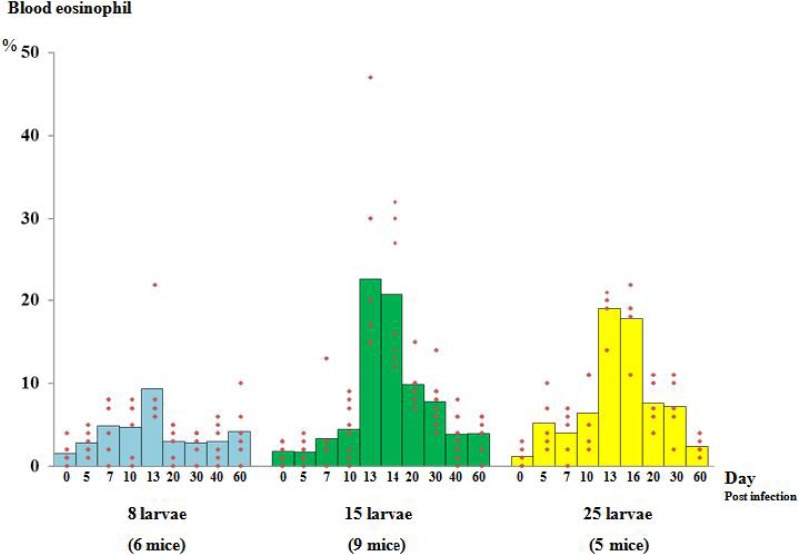
Percentage of blood eosinophil at different time intervals of mice infected with 8, 15 and 25 larvae

The percentage of blood eosinophils in mice prior to the infection ranged from 0%-4% (average 1.50%, + 1.38%). After infection, the blood eosinophils reached a peak at Day 13 PI. The highest percentage in this study was 47% from those infected with 15 AL3. There was significant difference between the 8 larvae infected group (9.33% + 6.25%) and the 15 larvae infected group (average 22.66% + 11.03%, *P*-value = 0.001) (Fig. 3).

Interestingly, when the higher infectivity dose (25 larvae) group was studied, a higher percentage of blood eosinophils were not seen (average 19.00% + 2.92%). There was a significant difference between the 8 and 25 larvae-infected animals (*P*-value = 0.001). However, there was no statistically significant difference between the 15 and 25 larvae- infected groups.

## Discussion

### Larva migration

Our study confirmed that the mouse is not a definitive host of *G. spinigerum* since all the worms recovered were advanced third stage larvae (Fig. 1). They were unable to mature to adulthood. Over the past decades, rodents (rat, mouse and rabbit) were used in experimental studies of the effectiveness of chemotherapy. These studies reported that the average worm recovery was only 40% (ranged from 13.33%-74.00%) (23, 29, 30). The most common reported finding of worm numbers found in humans was only 1-3 worms (1, 6–8, 31). We therefore used 8 larvae as a feeding dose with an estimate of at least 2-3 worms that could be recovered for study of larval migration. Previous investigators showed that all *G. spinigerum* larvae must first move from stomach to liver and they suggested that the liver was the first organ where worms would remain for a period of 7-14 days before eventually migrating to other sites (21–23). However, the first sacrificial dates in those studies were after 1-2 week PI. We studied worm recovery at different hours during the first day after oral infection (Table 1). Our results showed that during 3-5 hr PI, the worm could penetrate the stomach wall and freely migrate through many viscera. At 5 hr PI, worms could be recovered from esophagus, liver, lung, heart and diaphragm.

Our results concurred with other investigators who studied animals infected with other *Gnathostoma* species, i.e. *G. hispidum* and *G. nipponicum*. After infection, the larvae could move freely from stomach to many viscera. It was not necessary for the larvae to reach the liver as first destination (24–26). Our larvae could be found in the liver at every time point throughout the study with different numbers of the worms’ recovered. The average total worm found in our mice was 78.03% (ranged 45.58%-100%) which was higher than in previous observations. The regulation of helminth population in infected hosts is a complex process influenced by duration and dose of infection, host defense mechanism and other factors (32). Previous studies showed that parasites can migrate to various visceral organs including liver, lung, urogenital tract, central nervous system, eye and others (6, 7, 10, 12, 13, 15). We could not detect eye involvements in our mice and this agrees with findings of others in animal models of mice, rats and rabbits (21–23, 29, 30). We found subcutaneous larvae migration in only 3 mice among a total of 33. In addition, the larvae were first found in subcutaneous tissues on Day 15 PI (5.88%). Throughout the experiment, muscles were the major site of larval involvement (76.47%-86.67%) which was mostly found by Day 15 and 5 months PI.

Encysted larvae were detected in multiple tissues such as diaphragm, subcutaneous tissue, liver and others. They were enclosed by dense fibrosis with mild to moderate chronic inflammatory cell infiltration (Fig. 2). Larval encystment was also observed in the infected rabbits and rats by other investigators (22–24, 29, 30). In contrast, the high frequencies of subcutaneous migratory swelling (approximate 50% in infected individuals) with human gnathostomiasis, is well known. Gnathostoma worm encystment in humans has never been observed). (1, 6, 8, 31, 33, 34). We hypothesized that the larvae in humans keep migrating and move more superficially (subcutaneous tissue) in order to evade host defenses (31).

### Blood eosinophilia

Of the seven mice with high level of 30 larvae infection, five died on day 2-8 PI (data not shown). Autopsy showed severe disruption of liver parenchyma. Therefore, we reduced the doses to 8, 15 and 25 larvae for study of blood eosinophilia. Fluctuation of blood eosinophilia during the subsequent weeks was then observed. The peak in blood eosinophils in our infected animals seen was on day 13 PI. The highest percentage in blood eosinophils was 47% which was detected in the 15 AL3 infected group. However, the average percentage count was higher in the 15 larva infected group than in the 8 larva infected group. Different larval loads affect the amount of parasite protein for eosinophil stimulation. Rats infected with 3 larvae of *G. nipponicum* showed only 6% maximum blood eosinophilia (25). Surprisingly, when the higher infective dose (25 larvae) was used in our study, blood eosinophilia (average 19.00% + 2.92%) was not higher than in the 15 larvae infected group (average 22.66% + 11.03%). This may be due to a maximum point of responsiveness of hematopoietic progenitor cells or the bone marrow pluripotential stem cells to proliferation and differentiation to eosinophil (35). Eosinophilia induced by parasitic infection is dependent on interleukin-5 produced by Th-2 subset of CD4^+^ helper T cells (36). Excessive involvement of the Th-2 response can result in depression of immune cells which occurred in chronic and/or heavy dose of parasitic infections (37, 38).

Elucidation of mechanisms by which *G. spinigerum* induces low production of eosinophils with high parasite dose infected animals, could help to expand our knowledge and to advance understanding of the regulatory events governing eosinophil activation. However, after the blood eosinophilia reached a plateau, it gradually decreased and dropped to low levels within 2 months PI. Our findings suggest that a low eosinophil count does not indicate low parasite burden. This might be due to the encystment of worms, thus causing a low amount allergen for eosinophilic stimulation. In human gnathostomiasis, about 70% patients demonstrate blood eosinophils higher than 5% which may exceed 50% of circulating white blood cells (1, 8, 20, 31). Blood eosinophils decrease in patients only in cases after drug treatment or surgical removal of worms (8, 10, 31). The potential components which stimulated eosinophilia might be present in greater proportion during the invasive stage and decline in the encysted stage (39).

In conclusion, our finding indicated that after infection *Gnathostoma* larva can migrate freely to various viscera. Liver involvement was detected at 3 hr post infection throughout the study. We confirmed that mice are accidental hosts and larva encystment occurred. In contrast, humans do not experience worm encystment. In our study, high blood eosinophilia is seen in high dose-infected animals. The level in the blood eosinophilia reaches a plateau depending on the infective dose. In addition, a high blood eosinophil could occur during the invasive stage and drop to a low level when the parasites encysted. Mechanisms that stimulate encystment should be topic for further study and have practical value. It may not be the fact that larvae encysted in mice have made them poorer animal model for evaluation of drug treatment or other studies.
